# Educational assessment without numbers

**DOI:** 10.3389/fpsyg.2024.1399317

**Published:** 2024-10-02

**Authors:** Alex Scharaschkin

**Affiliations:** ^1^Department of Education, University of Oxford, Oxford, United Kingdom; ^2^AQA Education, London, United Kingdom

**Keywords:** theory and philosophy of measurement, psychometrics, educational assessment, van Fraassen, qualitative mathematics, concept lattice, fuzzy logic

## Abstract

Psychometrics conceptualizes a person's *proficiency* (or *ability*, or *competence*), in a cognitive or educational domain, as a latent numerical quantity. Yet both conceptual and empirical studies have shown that the assumption of quantitative structure for such phenomena is unlikely to be tenable. A reason why most applications of psychometrics nevertheless continue to treat them as if they were numerical quantities may be that quantification is thought to be necessary to enable *measurement*. This is indeed true if one regards the task of measurement as the location of a measurand at a point on the real number line (the viewpoint adopted by, for example, the representational theory of measurement, the realist theory of measurement as the discovery of ratios, and Rasch measurement theory). But this is not the only philosophically respectable way of defining the notion of measurement. This paper suggests that van Fraassen's more expansive view of measurement as, in general, *location in a logical space* (which could be the real continuum, as in metrological applications in the physical sciences, but could be a different mathematical structure), provides a more appropriate conceptual framework for psychometrics. Taking educational measurement as a case study, it explores what that could look like in practice, drawing on fuzzy logic and mathematical order theory. It suggests that applying this approach to the assessment of intersubjectively constructed phenomena, such as a learner's proficiency in an inherently fuzzily-defined subject area, entails recognizing the theory-dependent nature of valid representations of such phenomena, which need not be conceived of structurally as values of quantities. Finally, some connections are made between this “qualitative mathematical” theorization of educational assessment, and the application of techniques from machine learning and artificial intelligence in this area.

## 1 Introduction

The question of what it could mean to *measure* phenomena that form the basis of theory and debate in the human sciences, such as human attitudes, opinions, dispositions, or psychological or cognitive traits, has been a subject of critical enquiry since at least the mid eighteenth century (Michell, 1999). For example, the question of whether such phenomena could be *quantified* was contested by Reid (1849), even before a clearer definition of “a quantity” had been put forward by Hölder (1901).

This paper considers the question of measuring educational constructs, such as a learner's *ability*, or *proficiency*, or *competence* in a subject, field of study, or educational domain. Many educational tests and assessment procedures—some of them used to make high-stakes decisions about the test-takers—apparently produce, or claim to produce, numerical measurements of such properties, such that learners can be placed on a quantitative *scale* with respect to them. Psychometrics is the application of statistical methods to the study of psychological and educational phenomena. It relies on the particular mathematical characteristics of quantitative structures (in practice, the real numbers and vector spaces over the reals) to perform calculations and procedures that are used as the warrants for substantive conclusions, such as “how much” ability a student is estimated to have, or how to equate measurements of ability derived from different tests.

The paper argues that the reliance of psychometrics on quantitative structures is grounded in an assumption that *quantification* is necessary to allow *measurement*. It proposes, however, that psychological and educational measurement need not be reliant on numbers. It suggests that van Fraassen's (2008) account of measurement as a process whereby the measurand is located in an appropriate “logical space” is well-suited to serve as a foundation for an account of the measurement of educational phenomena such as students' abilities or competencies in a subject domain—phenomena that are arguably inherently “fuzzy” and multifaceted. Such a logical space *could* be the particular mathematical structure that uniquely characterizes the real numbers (a complete ordered field, in mathematical terminology), but it need not be.

The structure of the paper is as follows. Section 2 briefly outlines the approach to measuring cognitive and educational constructs, by assuming quantitative structure, that became standard in psychometrics over the twentieth century. It summarizes critiques of the quantity assumption, and argues that these critiques have sufficient conceptual and empirical weight to warrant a serious explanation of what an approach to psychological and educational measurement could look like if the assumption is set aside. Taking the example of summative educational assessment in particular, it suggests that in many cases construct validity may be better served by a more generalized view of measurement, of the kind proposed by van Fraassen (2008). Van Fraassen's approach is explained in more detail in Section 3.

Section 4 makes the discussion more concrete by comparing quantitative and qualitative measurement approaches for a toy example of an educational test. This is extended in Section 5 to a consideration of the practicalities—in particular, the computational complexity—of applying qualitative mathematical (fuzzy order-theoretic) methods to the kinds of test response data that arise in real practice. And since traditional methods of analysis of educational assessment data are increasingly being supplemented, or even supplanted, by the application of techniques from natural language processing, machine learning, and artificial intelligence (AI), Section 6 considers some of the connections between educational measurement and AI-enabled classification procedures. Finally, the concluding discussion in Section 7 poses some questions for further research. It concludes that it is worth pursuing further conceptual and technical development of non-quantitative measurement approaches in psychometrics, especially since, with the rapid rise and application of AI (e.g., von Davier et al., 2021), there is a risk that psychometrics is simply replaced with data science—with the loss of substantive theoretical content concerning construct definition and the design of valid measurement procedures. A way forward is for psychometrics itself to develop into a discipline that rests on quantitative measurement when it is appropriate, but does not exclude a broader view.

## 2 Quantification in psychometrics

### 2.1 Abilities as latent quantities

Psychometrics normally conceptualizes a learner's *ability* (or *proficiency*, or *competence*) in a domain as a latent numerical quantity, θ (Kline, 2000; van der Linden and Hambleton, 1997). For each learner, a value of θ is calculated from the observed data arising from an assessment (e.g., item response data). The “more θ” a learner has (the higher their value of θ), the “better at” the assessment construct they are taken to be (modulo some “measurement error”). That is to say, the relation of *betterness*, between learners, as to the different levels, states, or configurations of their abilities, is taken to be adequately captured by the relation of *order* (≥) between numerical values. Moreover, to allow a value of θ actually to be derived for each learner, the set of all possible θ-values is normally supposed not only to be totally ordered, but quantitative and continuous.^1^ Making these structural assumptions about the property of *ability* enables it to be treated as if it were a real number. Hence the whole array of statistical techniques whose mathematical validity depends on the metric and topological properties of the real numbers (such as factor analysis, item response theory, maximum likelihood estimation, etc.) can be applied to obtain numerical values that are taken to be *measurements of learners' abilities* in the cognitive or educational domain in question.

This paper will argue that one should not think of the “betterness” relation between learners, as to their proficiency in a particular educational domain, as a total order relation (a ranking), in general, but rather as a partial order.^2^ Sometimes the way in which the assessment construct is defined will allow learners to be ranked as to their proficiency with respect to that construct. In other cases, it may only be possible to infer, for some pairs of learners, that their proficiency states, or levels, are non-comparable (qualitatively different). This does not preclude the possibility of grouping learners together into “coarser” ordinal classes (such as examination grades), such that one can infer that those who “pass” are more proficient than those who “fail”, for instance. It just means that, within the “pass” category, there may be some learners whose proficiencies, although both of at least a “pass” level, may be different, and non-comparable. This argument is developed further in Section 4 below.

There is a literature that critically examines the plausibility of assuming quantitative structure for phenomena such as ability (for example, Michell, 2006, 2009, 2012, 2013; Heene, 2013; Kyngdon, 2011; McGrane and Maul, 2020, and from a broader perspective, Uher, 2021, 2022a). One focus of this has been what Michell (2012) calls the “psychometricians' fallacy”: the implicit leap that is often made, from maintaining that a property has a totally-ordered structure (that its possible values, states, or levels can be ranked, that is, placed on an *ordinal scale*, as described by Stevens, 1946), to treating it as if it had quantitative structure (as if its values formed an *interval* or a *ratio* scale, in Stevens' typology).

In some cases it is possible to test empirically whether a property whose values are ordered is plausibly likely to have the further structure required for it to be quantitative. This is discussed in Section 2.2.2. Yet at an even more basic level, one might question why a construct such as *ability* with respect to a given cognitive or educational domain (specified in a more-or-less precise way), should even be regarded as a property that necessarily ought to have a totally ordered structure. Must it be a phenomenon that only occurs in such a way that any one person's ability-state is always linearly comparable with (larger than, the same as, or smaller than) any other person's state? Uher (2022b) makes an analogous point with respect to the use of rating scales to “measure” the property of agreement.

If one considers the actual data upon which the inferences derived from educational testing procedures are based, then as Kane (2008) notes, “we are likely to have, at best, a partial ordering, unless we arbitrarily decide that some patterns [of item response] are better than others”. In practice, and as discussed further in Section 4, almost all psychometric approaches to working with such partially-ordered data do indeed involve making decisions about how to use the data to generate a total order (with each learner's score being their location with respect to this total order).

The question whether such decisions are indeed “arbitrary” (and if not, which one is best or most appropriate) hinges, again, on how the measurand—each respondent's ability in the domain in question—is conceptualized. This issue is well-described by Maul (2017, p. 60), who notes that

Any effort to construct a measure of an attribute will have trouble getting off the ground in the absence of a sufficiently well-formed definition of the target attribute, including an account of what it means for the attribute to vary (i.e., what meaning can be attached to claims about there being “more” or “less” of it, between and possibly within individuals) and how such variation is related to variation in the observed outcomes of the instrument (i.e., item response behaviour).

It is suggested in Section 3.2 that questions of this kind form part of what van Fraassen (2008) refers to as the *data model* for the target attribute. It is rather rare for psychometrics textbooks to devote much attention to these theoretical or conceptual issues, however. Often (e.g., Raykov and Marcoulides, 2011) it is stated that psychological and educational measurement is concerned with appraising how individuals differ with regard to hypothesized, but not directly observable, attributes or traits, such as intelligence, anxiety, or extraversion. It is assumed that these traits are in fact quantities (for instance Kline, 2000, p. 18) simply states that “the vast majority of psychological tests measuring intelligence, ability, personality and motivation … are interval scales”), and models are then introduced to relate them to observable data such as test or questionnaire responses in such a way as to enable the numerical latent trait parameters to be estimated, together with measures of precision such as standard errors—all conditional on the adequacy and plausibility of the model that has been assumed. Of course if the model is not adequate as a structural theory of the phenomenon itself, then results may simply reflect artifacts of the model (e.g., consequences—sometimes rather trivial tautologies—that follow from the metric structure of the real numbers), rather than corresponding to valid inferences with respect to the theory of the phenomenon.

Why should a phenomenon such as a learner's proficiency or competence in a particular domain be assumed to have the structure of a total order (let alone a quantity)? The reason probably goes back to a belief fundamental to the early development of psychometrics, that quantitative structure is necessary to enable measurement. For example, Thurstone (1928) claimed that

When the idea of measurement is applied to scholastic achievement, … it is necessary to force the qualitative variations [in learners' performances] into a quantitative linear scale of some sort.

If “the idea of measurement” entails *locating a measurand at a point on the real number line*, then “forcing” observed qualitative variations to fit a quantitative structure is an understandable approach to adopt (even if it raises questions about validity). Indeed two common theoretical frameworks for psychological and educational measurement—the representational theory of measurement, and Rasch measurement theory—could be construed as concerned with ways to “force” qualitative variation into quantitative form: the former by aiming to define conditions under which qualitative observations can be mapped into numerical structures; the latter by rejecting observations that do not fit an assumed quantitative model. These approaches are unpacked a little in the next section.

### 2.2 Theories of measurement

#### 2.2.1 The representational theory of measurement

Tal (2020), in his survey of the philosophy of measurement in science, describes the representational theory of measurement (RTM) as “the most influential mathematical theory of measurement to date”. Wolff (2020), in a recent structuralist account of quantity and measurement, calls it “arguably the most developed formal theory of measurement”. Michell (1990) claimed that it is “the orthodox theory of measurement within the philosophy of science”.

The canonical text on RTM (Krantz et al., 1971, p. 9) takes *measurement* to mean “the construction of homomorphisms (scales) from empirical relational structures of interest into numerical relational structures that are useful”.

RTM supposes that we are given an “empirical relational structure” (itself an abstraction of certain features of an “observed reality”). This structure consists of objects, relations between them, and possibly also ways of combining or composing them. For example in educational measurement contexts, we might take as objects students' responses to a writing task, and consider a binary relation ≽ of *betterness* as being of interest (as in “student *X*'s piece of writing is a better response to the task than student *Y*'s: *X*≽*Y*”). Or we might be interested in how parts of a test or assessment combine (via a binary operation •) to form an overall measure. For example, “correctly answering questions 3 and 4 demonstrates a higher level of proficiency than correctly answering questions 1 and 2”: *q*_3_ • *q*_4_≽*q*_1_ • *q*_2_. We might then wish to investigate whether these aspects of students' responses to tasks—this empirical relational structure—can be mapped to a numerical ordering or scoring system, in such a way that the structure is preserved (e.g., relative betterness between responses is mirrored by the relative magnitudes of the numbers assigned to those responses).

The idea is that if such homomorphisms can be shown to exist, then inferences in the numerical relational structure (normally taken to be the real numbers with the usual order relation ≥ and binary operations + and ·) provide warrants for conclusions in the substantive domain of the empirical relational structure. If, further, we posit that differences in the observed outcomes of an educational assessment procedure, such as the administration of a test or examination, are *caused by* differences in the configurations, between learners, of their “underlying proficiency”, then establishing a homomorphism between the empirical relational structure and the real numbers [i.e., establishing that the outcomes can be “placed on an interval (or ratio) scale”] serves to justify the assumption of quantitative structure for this assumed underlying proficiency trait, and hence to enable the measurement of each test-taker's proficiency by locating them at the point on the real line that corresponds to their level of proficiency.

#### 2.2.2 Qualitative relational structures and testing for quantity

The adequacy of RTM as a theory of measurement has been extensively critiqued (see, e.g., Michell, 1990, 2021; see also Luce and Narens, 1994), with commentaries noting that its abstract nature sidesteps the actual process of measuring anything, the construction of measuring instruments, and any discussion of measurement error. The merits of such critiques are not discussed further in this paper, because the position adopted here will be that of Heilmann (2015). Heilmann (2015, p. 789) does not assess RTM as a candidate for a theory of measurement, but rather as a collection of mathematical theorems: theorems whose structure makes them useful for investigating problems of concept formation. He proposes viewing theorems in RTM as

providing us with mathematical structures which, if sustained by specific conceptual interpretations, can provide insights into the possibilities and limits of representing concepts numerically

He regards RTM as studying not mappings from an empirical relational structure to a numerical relational structure, but rather from a *qualitative relational structure* (QRS) to a numerical relational structure. Taken in that sense, he argues, RTM can provide tools for testing the extent to which abstract concepts (captured or described as qualitative relational structures) can be represented numerically.^3^

Arguably, this is how RTM (including in particular the subset of RTM theorems that form the so-called theory of *conjoint measurement*: see Luce and Tukey, 1964) does in fact tend to be used in the literature exploring the plausibility of assuming quantitative structure for educational, psychological, or social measurands.

For example, Michell (1990) re-analyzed data collected by Thurstone (1927b) regarding judgements as to the seriousness of various crimes. Thurstone (1927a) claimed that his theory of *comparative judgement* enabled the construction of a *quantitative scale* for the measurement of seriousness of crime, by applying the theory to the outcomes of a collection of pairwise comparisons, in which subjects were repeatedly asked which of two crimes presented to them was the more serious. Michell (1990, p. 107) carefully stated the assumptions of Thurstone's theory, and demonstrated by applying results from RTM that “either seriousness of crimes is not a quantitative variable, or else some other part of Thurstone's theory of comparative judgement is false”.

van Rooij (2011) applied theorems from RTM to explore whether properties of objects, that manifest linguistically as adjectives with comparative degrees, can be represented numerically, what scale properties may hold for them, and hence whether inter-adjective comparisons (such as “*x* is *P*-er than *y* is *Q*”) can be meaningful. This is analogous to the vexed question, in educational assessment, of inter-subject comparison when it comes to setting and maintaining qualification standards (see, e.g., Newton et al., 2007; Coe, 2008).

Karabatsos (2001, 2018), Kyngdon (2011), Domingue (2014), and Scharaschkin (2023) applied theorems from RTM to the question of testing whether psychometric attributes comply with requirements for quantitative structure, combining the RTM results with a stochastic approach to address expected “measurement error” in most measurement scenarios with reasonable numbers of test-takers and test items. Domingue found that the results of a well-known test of reading showed that it was highly implausible that reading proficiency was a quantitatively-structured variable. Scharaschkin found that the results of a test of physics for school-leavers did not support the assumption of quantitative structure for a hypothesized “physics proficiency” construct. On the other hand, he found that the results of a similar test of economics were approximately consistent with an assumption of quantitative structure.

None of these applications require assuming the validity or adequacy of RTM as a substantive theory of measurement—indeed, Michell (2021) explicitly rejects it. Yet they do shed light on the extent to which qualitatively-structured data can be treated *as if* it were a manifestation of quantitatively-structured latent traits, and provide empirical evidence that it is not always valid to do so.

This is relevant to the practice of educational assessment and test construction because most practitioners and test developers probably do work within a pragmatic “as if” framework, as summarized by Lord and Novick (1968, p. 358):

Much of psychological theory is based on trait orientation, but nowhere is there any necessary implication that traits exist in any physical or physiological sense. It is sufficient that a person behave as if he were in possession of a certain amount of each of a number of relevant traits and that he behave as if these amounts substantially determined his behaviour.

Some of the ways in which theories of cognition have been more directly incorporated into the use of quantitative latent variable modeling, and their relation to the ideas considered in this paper, are discussed further in Section 5.4.

#### 2.2.3 Rasch measurement theory

Psychometrics conducted in the Rasch measurement tradition (Andrich and Marais, 2019) takes the view that measurement is only meaningful for quantitative phenomena. Thus, if a putative measurement procedure such as an educational or psychological test yields results that are inconsistent with a underlying quantitative variable, then the procedure is not, in fact, *bona fide* measurement, and requires modification. In practice this means modifying tests by deleting or changing items until a sufficiently good fit to the Rasch model is obtained.^4^

So rather than trying to find a model that fits the data that has been obtained from the administration of a test, the Rasch measurement approach is to try to make the data fit the model. Modifying the measurement instrument to achieve this may come at the cost of severely constraining the theory of (or, in the terminology of Section 3.2, the relevant data model for) the substantive phenomenon or construct of interest. It might be that the construct cannot be sufficiently constrained or re-defined without significantly departing from its underpinning theory of value. In an educational assessment context, this would be the case if making such changes to the assessment instrument would compromise construct validity: the assessors' understanding of what constitute the key attributes of proficiency in the given domain, and how relatively better/worse/different states of proficiency would present with respect to these attributes. In such cases the choice would seem to be either to abandon the idea of measuring the construct at all, or to abandon the restriction of measurement to locating measurands within solely quantitative mathematical structures. This paper explores the latter option.

#### 2.2.4 Measurements as ratios

Michell (1999) traces the evolution of the concept of measurement in psychology since the publication of Fechner's *Elemente der Psychophysik* in 1860. He bemoans the movement away from the conceptualization of measurement that had become standard in nineteenth century physics, namely (Michell, 1999, p. 14) “the discovery^5^ or estimation of the ratio of the magnitude of a quantitative attribute to a unit (a unit being, in principle, any magnitude of the same quantitative attribute)”. In other words, as elementary physics texts still state, physical quantity = real number × unit, where the real number is the measurement of the physical quantity.

Michell notes (p. 19) that “according to the traditional understanding of measurement, only attributes which possess quantitative structure are measurable. This is because only quantitative structure sustains ratios”. He argues that, this being the case, it is incumbent on psychometricians to investigate whether the phenomena they study do, in fact, have quantitative structure, before applying statistical models that assume it. Since in practice this is almost never done, his claim is that, for the most part, “psychometrics is built upon a myth” (Michell, 2012). Once again, the choice appears to be to accept the constraints of the “traditional understanding of measurement”, or to explore whether psychometrics could benefit from engagement with a more expansive conceptualization of what it means to measure something. The next section considers such a viewpoint.

## 3 van Fraassen's account of measurement

### 3.1 Basic principles and relevance to psychometrics

Bas van Fraassen's (2008) *Scientific Representation: Paradoxes of Perspective* is an empiricist structuralist account of measurement and representation in science. This stance eschews debate about the ontological status of the phenomena or reality that scientific theories describe, and concerns itself rather with elucidation of what van_fraassen argues is the key aim of developing and testing such theories, namely their empirical adequacy. van Fraassen (2008, p. 2) claims that “measuring, just as well as theorizing, is representing … measuring *locates* the target in a theoretically constructed logical space”. To be more precise (p. 164),

measurement is an operation that locates an item (already classified in the domain of a given theory) in a logical space (provided by the theory to represent a range of possible states or characteristics of such items).

A key point here is the theory-relatedness of measurement procedures. Echoing Maul's (2017) requirements, quoted in Section 2.1, for a “well-formed definition of the target attribute” as fundamental to psychometric measurement, van Fraassen suggests (p. 166) that “once a stable theory has been achieved, the distinction between what is and is not genuine measurement will be answered *relative to that theory*”.

It is argued in Section 4 that a candidate theory for the phenomena (proficiency or competence in a domain) that form the subject matter of educational measurement, is a description of what constitutes betterness between learners' possible states or configurations of proficiency in a given domain. “Betterness”—which, as noted in Section 2, may be a more general order relation than a simple ranking—has to be defined in terms of criteria that may, in general, be manifested with *fuzzy degrees of truth* in the responses of learners to tasks that have been designed to provide information about their proficiency in the domain in question.

van Fraassen considers several measuring procedures in classical and quantum physics (p. 157–172 and 312–316), and concludes (p. 172) that they are all “cases of grading, in a generalized sense: they serve to classify items as in a certain respect greater, less, or equal. But … this does not establish that the scale must be the real number continuum, nor even that the order is linear. The range may be an algebra, a lattice, or even more rudimentary, a poset”. In fact, Section 4 below considers the case of lattices as logical spaces for educational measurement procedures.^6^

It is worth exploring how van Fraassen's approach could be applied to educational measurement for at least two reasons. Firstly because, as discussed in Section 2.2.2, the mathematically necessary conditions for a learner's proficiency in a given educational domain to have the structure of a quantity often do not hold; and it is not possible to massage the assessment instrument to make them hold without loss of construct validity. In such cases, it would arguably be inappropriate to theorize the construct as quantitative, and hence its measurement as location *on the real line*, rather than in some other, theory-relevant, logical space.

Secondly, the approach of thinking about educational assessment constructs in terms of fuzzy criteria of value (what will count as creditworthy, or indicative of good/bad performance, in relation to what particular domain content) is what *actually happens in practice*, when subject domain experts develop and administer at least one kind of high-volume, high-stakes, educational assessment procedure, namely the public examinations taken by school pupils aged 16 and 18 in the UK. This brings us to a consideration of what van Fraassen calls *data models*.

### 3.2 Data and surface models

Measurements arise from the results of procedures designed to gather information about a phenomenon of interest. As noted in Section 2.2.2, these entail selective attention to specific features that are deemed to be relevant. That is to say, measuring a phenomenon involves collecting data structured in a specific way. van Fraassen (2008, p. 253) calls such a structure a *data model* for the measurand in question. He notes that

A data model is relevant for a given phenomenon, not because of any abstract structural features of the model, but because it was constructed on the basis of results gathered in a certain way, selected by specific criteria of relevance, on certain occasions, in a practical experimental or observational setting designed for that purpose.

In educational measurement we have gathered in a certain way (via an assessment procedure such as a test), selected by specific criteria of relevance (construct-relevant criteria: Pollitt and Ahmed, 2008) on certain occasions (at a particular point or points in time), in a practical setting designed for that purpose (e.g., the rules of administration and physical requirements for conducting an examination).

In the case where the test consists of a sequence of dichotomously-scored items *I*: = {*i*_1_, …, *i*_*n*_} administered to a collection *L*: = {*l*_1_, …, *l*_*m*_} of learners, we can think of this measurement setup as a map *V*:*L*×*I* → {0, 1} that assigns to each instance of a learner encountering an item the valuation 1 if they answer it correctly, and 0 if they answer it incorrectly. Equivalently, we can think of the information collected by the assessment procedure as organized in an *m*×*n* matrix whose (*m, n*) entry is *V*(*l*_*m*_, *i*_*n*_). There is, however, more structure entailed by the “betterness” ordering within each item (namely that “1” is better than “0”) than immediately stands out from simply viewing the data as a table. As discussed in Section 4.2, the totality of the results-plus-valuation-system can be viewed as a lattice (the so-called *concept lattice* for the data table)—and it is suggested in Section 4 that such lattices (generalized to incorporate fuzzy valuations if necessary) form the natural data model for the phenomena that educational measurement procedures, such as tests and examinations, aim to measure.

van Fraassen (2008, p.253) describes constructing a data model as “precisely the selective relevant depiction of the phenomena *by the user of the theory* required for the possibility of representation of the phenomenon.” In the context of educational testing, the proficiencies being studied are proficiencies or competencies *with respect to* a specified domain (such as “high school chemistry”, or “A level French”). What “good performance” or “good demonstrated attainment” looks like in these domains (and hence what would count as evidence of better or worse levels, or states, or configurations, of learners' *proficiencies*) is always subject to a prevailing understanding or agreement as to what potential aspects of the domain are chosen as relevant for discrimination between learners' performances as to their quality. In other words, the criteria for creditworthiness of candidates' responses to tasks in an assessment can be regarded as the selective relevant depiction of the phenomenon of interest, by those members of the competent authority (the “users of the theory”) who design, administer, and grade the tests. For that reason, concept lattices derived from the outcome data from the tests, that encode the relationship between learners and the assessment criteria, are appropriate data models.

In practice, van Fraassen (2008, p.167) notes that data models may be “abstracted into a mathematically idealized form” before empirical or experimental results are used to explore theories or explanations, or for substantive purposes. He gives the example of a data model consisting of relative frequencies, which is “smoothed” such that frequency counts are replaced with probabilities. An idealized or simplified version of a data model is called a *surface model* for the phenomenon in question. Surface models are considered further in Section 5.

## 4 Theories of constructs: comparing item response theory and fuzzy concept analysis

### 4.1 A small example

Table 1 shows results from an assessment that generates data on each of three items (or attributes) {*i*_1_, *i*_2_, *i*_3_} for six learners {*l*_1_, …, *l*_6_}. Here 0 means “not demonstrated”, $\frac{1}{2}$ means “partially demonstrated”, and 1 (or $\frac{2}{2}$) means “fully demonstrated”.

**Table 1:** Data from a test.

**\**	***i*_1_**	***i*_2_**	***i*_3_**
*l*_1_	0	$\frac{1}{2}$	$\frac{1}{2}$
*l*_2_	$\frac{1}{2}$	$\frac{1}{2}$	$\frac{1}{2}$
*l*_3_	1	1	$\frac{1}{2}$
*l*_4_	$\frac{1}{2}$	$\frac{1}{2}$	$\frac{1}{2}$
*l*_5_	0	$\frac{1}{2}$	0
*l*_6_	$\frac{1}{2}$	1	1

**Figure 1:**
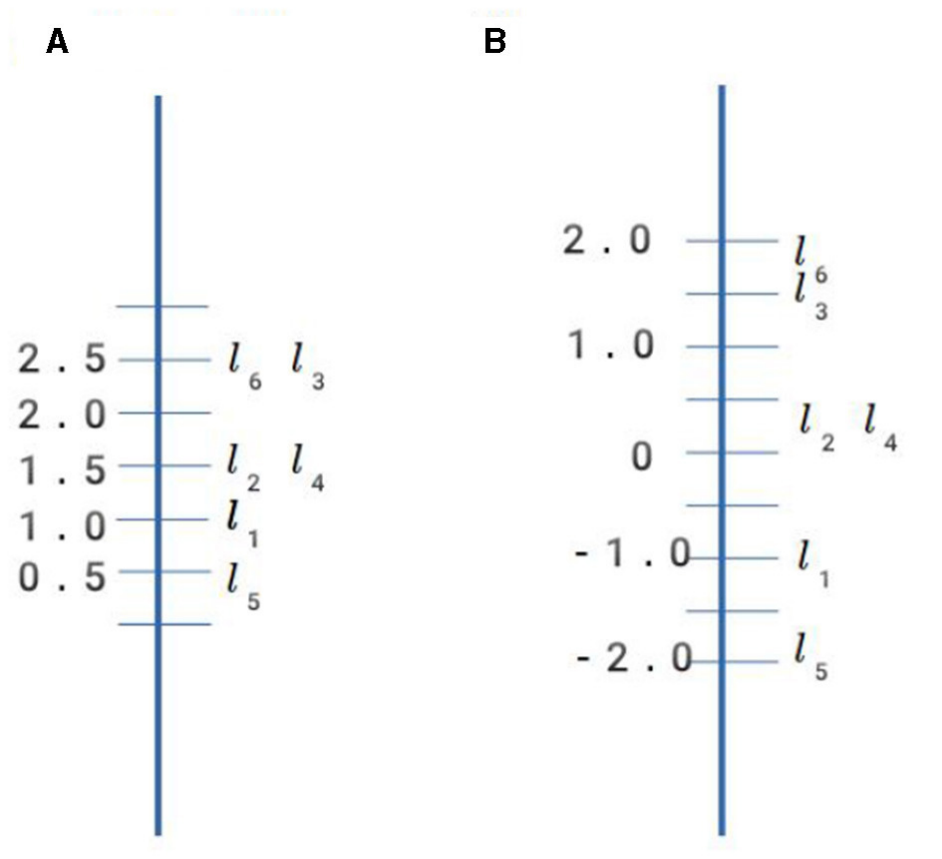
IRT-derived proficiency measures. **(A)** Sum score. **(B)** Latent variable score.

A traditional psychometric approach to analyzing this kind of data would be to treat each learner's results from the assessment as a vector in ℝ^3^, and each learner's proficiency measure as a quantity (a point in ℝ). For example, we could treat the label for each item response category as a number, and add them to get a total score for each learner. This orders learners, with respect to proficiency, equivalently to fitting a Rasch model (a 1-parameter item-response model), since total score is a sufficient statistic for estimating proficiency in this model. Or we could do a principal components analysis and take the projection of each learner's item-response vector onto the component that accounts for the most variance as their proficiency measure (this is equivalent to fitting a 2-parameter item-response model: see Cho, 2023). Doing so for the data in Table 1 yields three components of which the first accounts for 72% of the variance in outcomes, with the other two accounting for 19 and 9%, respectively. We could therefore take the loading (projection) of each learner's results onto the first component as their score on an “underlying” quantitative variable that represents the assessment construct reasonably well. Figure 1 shows how learners' proficiency measures differ depending on the approach taken.

**Figure 2:**
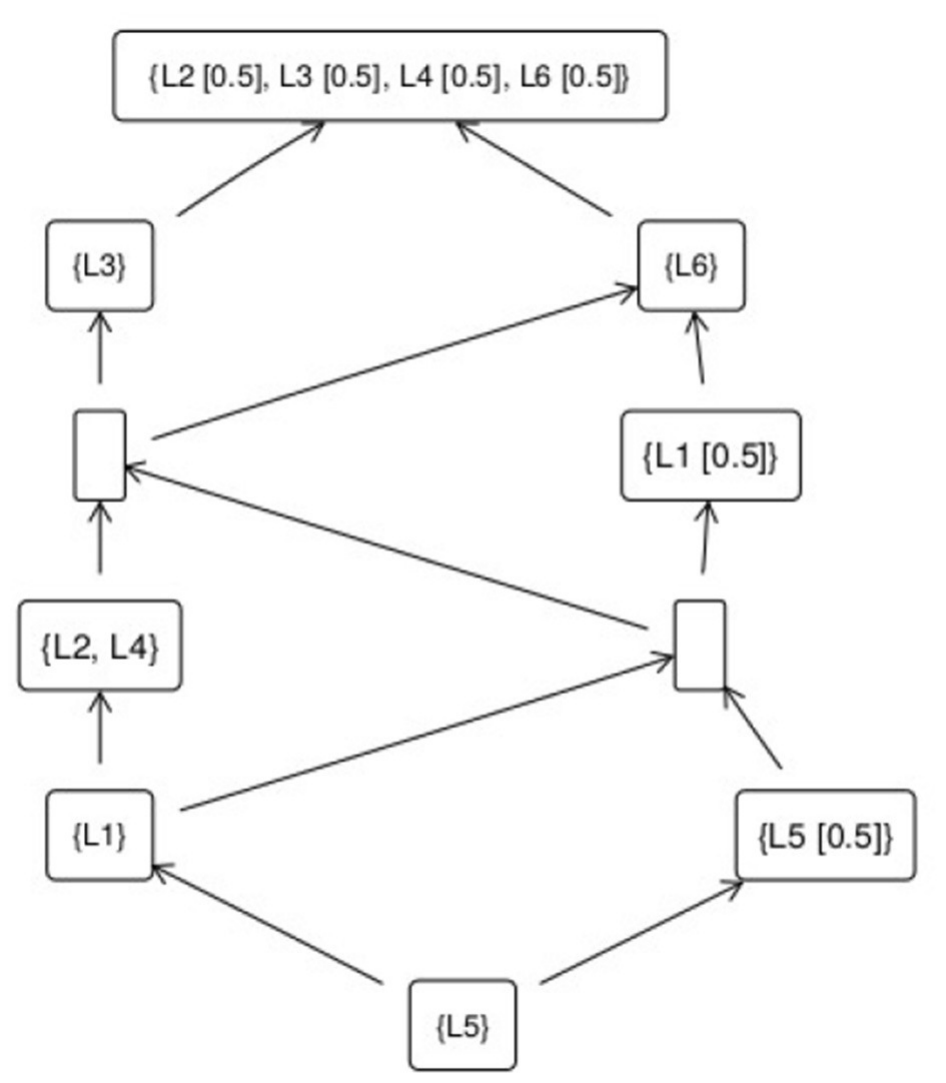
Fuzzy concept lattice for assessment data.

However, in view of the problems associated with assuming quantitative structure for proficiency discussed in Section 2.1 (tantamount, in Section 3.2's terms, to replacing the data model with a radically different surface model), let us consider a non-quantitative approach. If we take each learner's test response not as a vector of numbers, but rather a vector of ordered labels, then the observed data can be characterized as a collection of partially-ordered nodes: a network of “betterness” relations between nodes. In this data model, shown in Figure 2, each node is a *type of performance* on the assessment.

Each type of performance is defined by a collection of *attributes*, that *characterize* it; or (dually) by a collection of *learners*, who *demonstrate* it. The boxes in Figure 2 are the different types of performances on the test. The best performance is at the top of the diagram, and the worst performance at the bottom. Attributes, and learners, may belong to nodes to a *fuzzy degree*. Thus learner 5 belongs to (demonstrates) the lowest type of performance completely (to degree 1). Learners 2, 3, 4, and 6 all demonstrate the highest type of performance to degree 0.5.

*Better* types of performance are characterized by showing *more* attributes (and, dually, are demonstrated by *fewer* learners) than worse types of performance. An arrow from a box *A* to a box *B* means that *B* is a better performance than *A* (and by extension better than any performance *C* such that there is a connected path from *C* to *A*). If there is no path between two types of performance, then they are not comparable. Locating a learner (measuring their proficiency), with respect to this data model for the construct which the three-item test aims to assess, then means finding the “highest” node that they belong to in the network. This intuitive description is made more precise in the following section.

### 4.2 Formal concept analysis and proficiency measurement

Formal concept analysis (Ganter and Wille, 1999; Carpineto and Romano, 2004) is an important development of mathematical order theory that has been applied extensively to fields such as linguistics, political science, information sciences, medicine, and genetics. A recent application (Bradley et al., 2024) is to elucidating the mathematical representation of structure in large language models such as ChatGPT, discussed briefly below in Section 6. It can be thought of as a way of making explicit the information structure that is implicit in a matrix—such as that in Table 1—which relates objects to attributes (or learners to test items). It provides methods to extract the concepts and implications that can be deduced from such data, and introduces a logic to reason and infer new knowledge.

Consider first the case of measuring proficiency in a domain by administering an *n*-item test to *m* learners, where each item is dichotomously scored, i.e., for each learner *l* and item *i*, it is either the case that *l* answered *i* correctly, or that *l* did not answer *i* correctly. Given a subset of learners *L*_1_: = {*l*_1_, …, *l*_*k*_}, let *I*_1_: = {*i*_1_, …, *i*_*j*_} be precisely those items that all learners in *L*_1_ got correct. Then the pair (*L*_1_, *I*_1_) is an instance of a *formal concept* present in the data. *L*_1_ is called the *extent* of the concept, and *I*_1_ is called its *intent*. We can equally well start with a subset *I*_2_: = {*i*_1_, …, *i*_*p*_} of items, and then form the concept (*L*_2_, *I*_2_), where *L*_2_ is precisely the set of learners who got all items in *I*_2_ correct.

The collection of all formal concepts extracted from a matrix or data table simply restates the information present by virtue of the way the data is structured due to the choice of attributes (test item responses, in this example), and the ordered valuations chosen for attributes (just the two categories 1 ≥ 0 in this case). However, it makes this structure more apparent (and graphically representable, as in Figure 1) because concepts are (partially) *ordered* via the set-theoretic notion of inclusion. A concept (*L*_1_, *I*_1_) is *more general* than a concept (*L*_2_, *I*_2_) if *L*_1_⊇*L*_2_ (or equivalently, if *I*_1_⊆*I*_2_). The most general concept is the one that has the largest extent (and smallest intent). In test performance terms, the most general concept corresponds to the bottom, or worst, performance: because every other performance has a larger intent (entails more correct items). Similarly, the least general concept (with the smallest extent and largest intent) corresponds to the top, or best, level of performance.^7^

We can think of formal concepts as different ways of performing on the test (i.e., different ways of exhibiting proficiency in the subject domain). Each type of performance—or exhibition of proficiency—can be described *extensively*, by showing the learners who demonstrated it. Or it can be described *intensively*, by showing the item-profiles that characterized it. These two modes of presentation correspond to different ways of training “measuring instruments” (traditionally, human judges; more recently machine-learning methods such as neural nets) to recognize what good/bad performance (high/low proficiency) looks like. One can either give *examples* of a certain kind of performance, until an assessor can correctly classify new instances, or one can give *descriptions* of that kind of performance (in this case, the relevant profile of item responses), to enable new instances to be classified (measured) correctly.^8^

For a small educational measurement procedure of this kind (small in terms of the number of items/tasks/relevant attributes on which data is collected, as well as small in terms of the number of subjects to which it is administered), the qualitative equivalent of a quantitative score is a learner's location in the concept lattice: the highest concept, in the partial order, to whose extent they belong. This level of proficiency is described, not as a numerical “amount” (location on a line), but rather by the intent of the relevant concept: the actual items they mastered (or, more generally, the construct-relevant attributes their performance demonstrated). For larger (more realistically sized) assessments, the concept-lattice data-model becomes too granular, as shown in Section 5, and we develop a notion of “prototypical” kinds of performances at a manageable number of levels, such that each learner's level, or state, of proficiearency can be described approximately in terms of its qualitatively closest prototype.

Before moving on to that discussion, it is necessary to consider the question of the fuzziness of the criteria that structure data models in many educational measurement procedures.

### 4.3 Truth degrees and fuzzy concepts

#### 4.3.1 Assessment results as truth degrees

Table 1 illustrates a situation that often obtains in educational assessment. Learners are given tasks, such as questions on a test, and they may be successful in engaging with them *to a certain degree*. The outcome of a learner's interaction with an item is not necessarily captured by the crisp dichotomy of {correct, incorrect}.

The usual way of dealing with this in psychometric models is to model response categories for polytomous items as a sequence of threshold points on a latent quantitative continuum. A learner's response is in a higher category if it results from their proficiency-state being higher than, but not otherwise different from, a learner whose response is in a lower category. Differences in proficiency must be conceived of as differences in degree, not in kind. Yet as Michell (2012, p. 265) notes, in the context of mathematics tests, “the differences between cognitive resources needed to solve easy and moderately difficult items will not be the same as the differences between resources needed to solve moderately difficult and very difficult mathematics items. This observation suggests that abilities are composed of ordered hierarchies of cognitive resources, the differences between which are heterogeneous.”

An alternative approach is to start by the viewing the dichotomous situation as providing information about learners' performances in the form of *propositions* of the form “learner *l* answered item *i* correctly”.^9^ This proposition is true just in case the (*l, i*) entry in the data table arising from the assessment is 1. So we can think of the entries in the table as truth values (with 0 meaning false and 1 meaning true).

It has long been recognized that, in situations in which there is inherent fuzziness, vagueness, or semantic uncertainty in concepts, bivalent logics, in which the only possible truth values for a proposition are {false, true} can be unduly restrictive (see e.g., Goguen, 1969; Goertz, 2006; Bělohlávek et al., 2017). *Fuzzy logic* (Hajek, 1998; Bělohlávek et al., 2017) allows propositions to have truth values drawn from ordered sets of *truth degrees*, that can be more extensive than {false, true}.

Thus we can view the example in Table 1 as providing information about propositions with three truth-degrees, that we could label $\left\{0,\frac{1}{2},1\right\}$, or {false, partially-true, true}. For example, it is false that learner *l*_1_ demonstrated attribute *i*_1_ (or we could say, she demonstrated it to degree 0), and it is partially-true that she demonstrated attribute *i*_2_ (she demonstrated it to degree $\frac{1}{2}$).

When the outcomes of educational measurement procedures are not completely and crisply dichotomous with respect to all the construct-relevant attributes about which information is collected, the concept lattice for the resulting matrix of fuzzy truth values is itself fuzzy. Objects and attributes belong to concepts with degrees of truth, rather than crisply. In the concept lattice in Figure 2, the label “0.5” after a learner-identifier means that learner belongs to the concept (i.e., has demonstrated that type or level of performance) to degree $\frac{1}{2}$).

Although a discussion of the concept of “measurement error” in psychological testing and educational assessment would take us beyond the scope of this paper, it may be worth clarifying, for the avoidance of doubt, that the application of fuzzy logic in this context is not simply an alternative to using probability theory. Probability is a tool that can be used to study (epistemic) *uncertainty* (the lack of precision that arises from incomplete or poor information), whereas fuzzy logic is a tool that can be used to study (ontological) *vagueness* (the inherent fuzziness, or necessary inexactness, of concepts like “proficiency” in a certain domain). Erwin Schrödinger, when considering what the development of quantum mechanics meant for the measurement of physical phenomena, distinguished these two facets when he noted (Trimmer, 1980; p. 328) that “There is a difference between a shaky or out-of-focus photograph and a snapshot of clouds and fog banks”.

The statement “Mary has a fairly good understanding of physics” is vague but certain, whereas “Mary will pass the physics test tomorrow” is precise but uncertain. Working with propositions such as the former (i.e., deploying what Goguen, 1969 calls a “logic of inexact concepts”) is core to educational assessment, because of the contestable and intersubjective nature of educational constructs, discussed further in Section 7.2.

#### 4.3.2 Truth degrees and quantities

Buntins et al. (2016) apply fuzzy logic to psychological tests in a somewhat different way to that proposed here. They take the view that scores obtained from a test should not “refer to latent variables but to the truth value of the expression ‘person *j* has construct *i*”, where a *construct* is defined by a collection of relevant *attributes*, each of which may be *possessed* by a test-taker to a certain degree, and each of which may be *relevant* for the construct to a certain degree. Modeling truth degrees as real-valued quantities in the interval [0,1], they present an algorithm for aggregating them across attributes to arrive at an overall score for each learner: the truth value of the proposition “this learner has the construct”. They are careful to distinguish the semantic vagueness of a construct definition (recognized in the use of fuzzy truth values) from the idea of “measurement error”.

Buntins et al. claim that this approach “neither relies on latent variables nor on the concept of [quantitative] measurement”. However, they do state it is arguable that “although there is no measurement theory involved in the … formalism, the application to actual test behavior does presume item answers to be assessed on an interval scale level”, because “test answers have to be real numbers between 0 and 1, reflecting the subjective truth-values of the corresponding attributes for the tested person … However, these only refer to the item level and do not extend to theories about latent variables.”

In fact truth degrees do *not* have to be real numbers between 0 and 1. What is required is that they have a way of being compared with each other—that is, an order structure (which could be a partial order)—and way of being combined with each other. In general these requirements are met by taking them to have the mathematical structure of a so-called complete residuated lattice (Hajek, 1998). Further work on conceptualizing truth degrees—and especially what that means for empirically eliciting them—is important, as touched on in Section 7, but beyond the scope of this paper.

Buntins et al. see their approach “not as opposed to psychometric theory but tr[ying] to complement it with an alternative way to conceptualize psychological tests”. By contrast, the approach presented in this paper is suggested not as an alternative to, but an extension of, psychometric theory: one in which quantitative measurement forms an important, but special, case of a more general measurement framework.

#### 4.3.3 Fuzzy relational systems

In summary, the argument in this section is that in general, educational assessment procedures that aim to measure constructs such as proficiency, ability, or competence in a fuzzily-defined domain, generate *fuzzy relational systems*: matrices of truth-values for propositions of the form “learner *l* has demonstrated construct-relevant attribute *i*”. As data models, these are equivalent to fuzzy concept lattices: partially-ordered hierarchies, or networks, of types of performance on the assessment, that are discriminable with respect to these construct-relevant attributes. The next section considers whether these data models can provide insight for realistically-sized assessments.

## 5 Practicalities of educational assessment with non-quantitative data models

### 5.1 Granularity of data models

**Figure 3:**
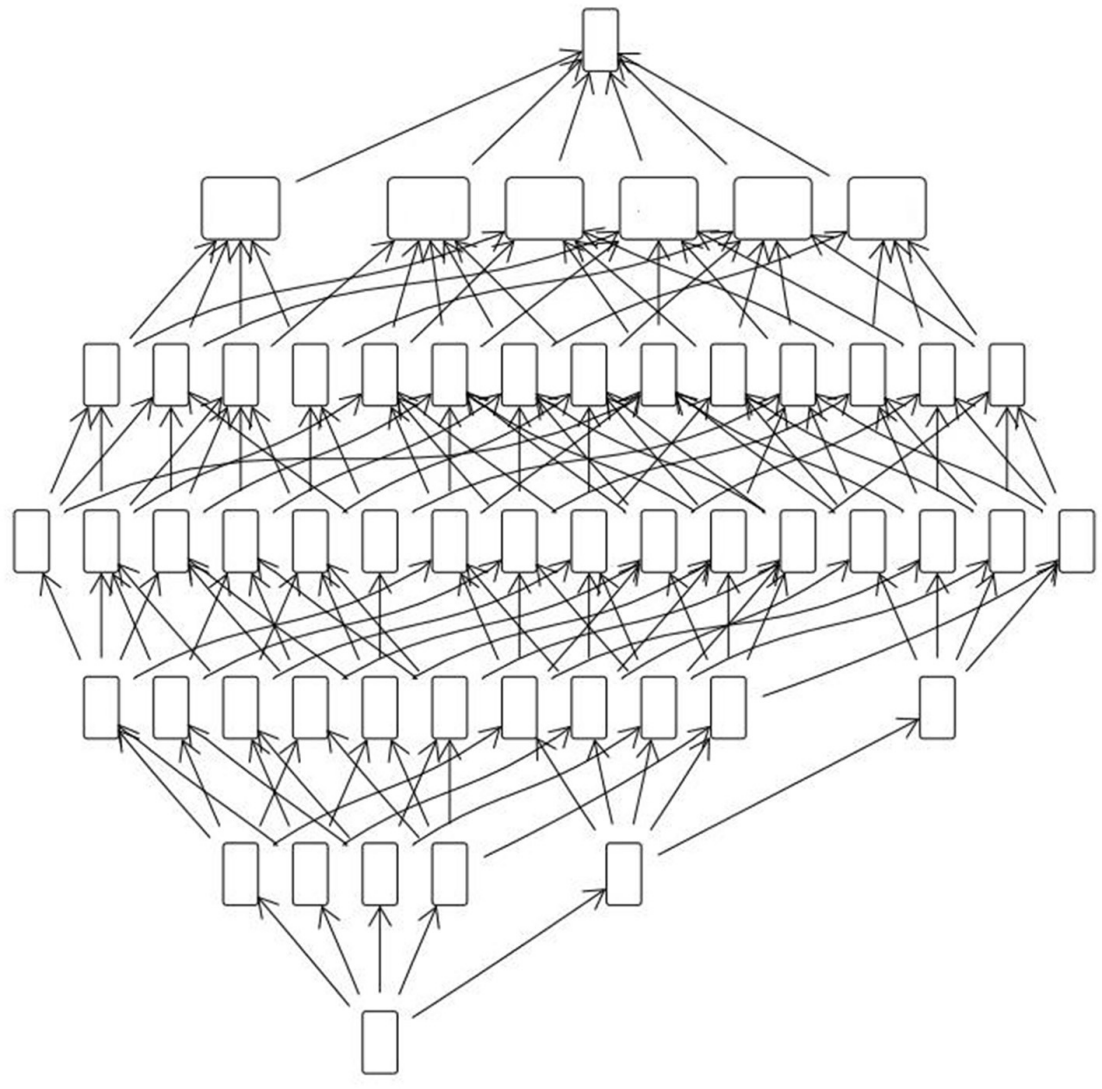
Concept lattice for a 5-item test with 100 learners.

**Figure 4:**
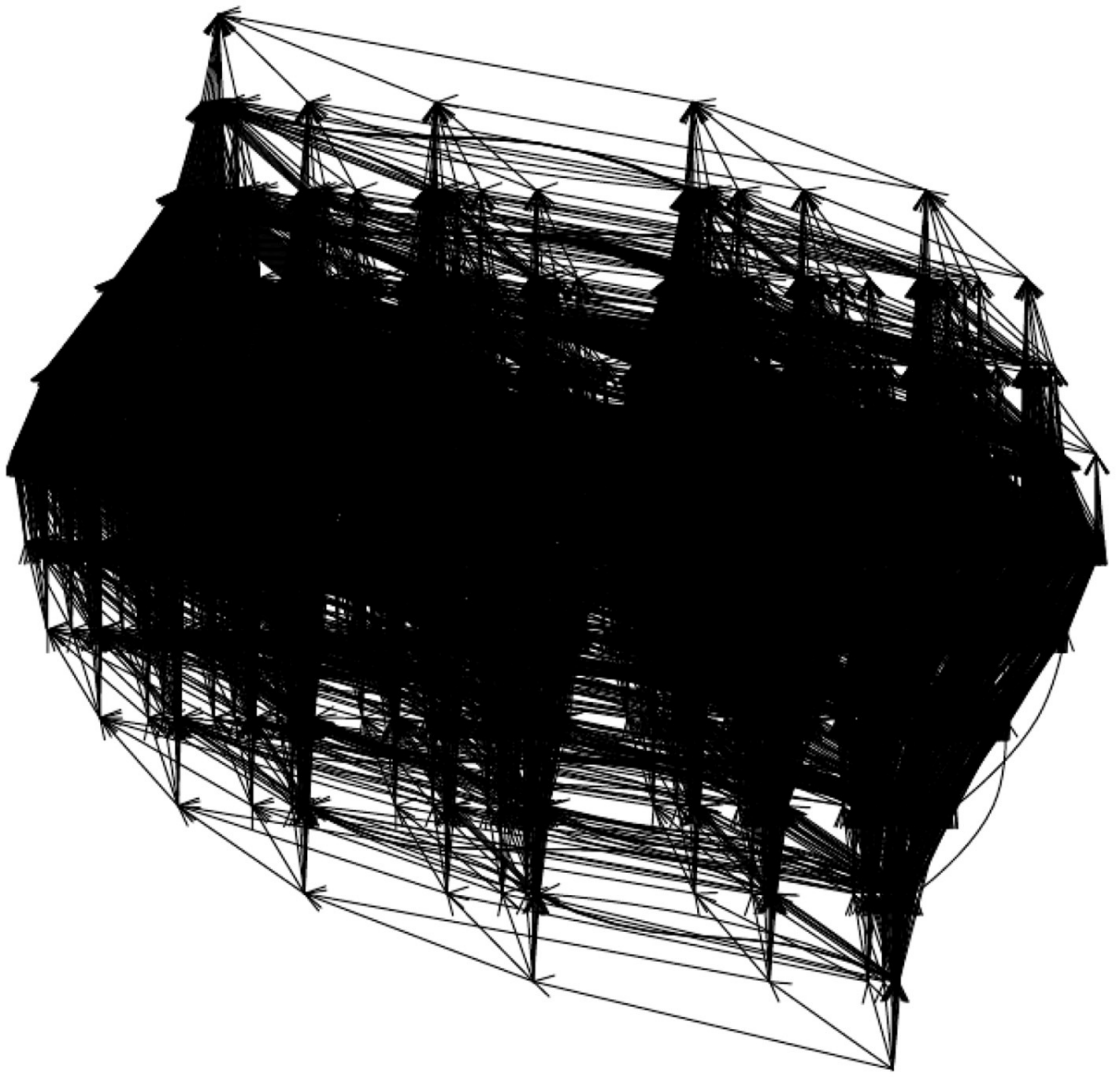
Concept lattice for a 12 item test with 200 learners.

An issue with data models of the kind discussed in the previous section is that their combinatorial complexity increases geometrically with the numbers of learners and construct-relevant attributes of performance (or test items) involved. Figures 3, 4, for instance, show the concept lattices for subsets of outcomes of a physics test.^10^ with increasing numbers of learners and attributes. Clearly the information here is too granular to be useful, and we need to simplify or “smooth” it in some way.

For quantitative data models, where learners' test responses as thought of as vectors in *n*-dimensional Euclidean space, the analogous granuarity-reduction is often performed using latent variable models that aim to find a *k*-dimensional subspace with *k*<*n* (often a one-dimensional subspace, i.e., a line) that is oriented in such a way as most closely to approximate the direction of most of the variation between the positions of these points (possibly subject to some other constraints as well, for certain factor-analytic models: see Bartholemew et al., 2008). Each learner's latent-variable score is then the projection of the vector that represents their test performance onto this subspace. Calculating these scores entails factorizing the (transpose of the) matrix *Z* of normalized test scores. If there are *m* learners and *n* test items, then the *n*×*m* item-by-learner matrix *Z*^*T*^ is factorized into the product of a *n*×*k* item-by-factor matrix *L* and a *k*×*m* factor-by-student matrix *F*, plus some error: *Z*^*T*^≈*LF*. Then using standard results in linear algebra, it can be shown (e.g., Reyment and Jöreskog, 1993) that the factors are the eigenvectors of the covariance matrix *ZZ*^*T*^.

### 5.2 Factorizing qualitative matrices

Bělohlávek (2012) studied the question of factorizing a matrix of fuzzy truth values. Now the matrix product is no longer defined in terms of operations on quantities, but rather in terms of operations on truth values.^11^ Let *M* be an *m*×*n* matrix arising from an educational measurement procedure conceptualized as in Section 4.3, so that *M*_*ij*_ is the degree to which learner *i* displays attribute *j*. By analogy with the quantitative case, consider an approximate factorization of *M* into a *m*×*k* learner-by-factor matrix *A* and a *k*×*n* factor-by-attribute matrix *B*, i.e., *M*≈*A* ◦ *B*. The key theorem in this case, due Bělohlávek (2012), is that *the factors are particular formal concepts* from the concept lattice for *M*. That is, “picking out key concepts” (particular types of learners' responses to the assessment) is equivalent to “logically factorizing” the matrix of truth-degrees that is the outcome of the measurement procedure.

The factors are the (extents and intents) of specific concepts in the concept lattice for *M*. The intuition is that, with *M*_*ij*_ = *A*_*ip*_ ◦ *B*_*pj*_:

- *A*_*ip*_ is the degree to which learner *i* is an example of (in the extent of) factor *p*;
- *B*_*pj*_ is the degree to which attribute *j* is one of the manifestations of (in the intent of) factor *p*;
- *M* = *A* ◦ *B* means: learner *i* displays attribute *j* if and only if there is a factor (formal concept) *p* such that *i* is an example of *p* (or *p* applies to *i*); and *j* is one of the particular manifestations of *p*.

Thus, the qualitative analog of projecting a Euclidean space onto a lower-dimensional subspace consists in picking out certain points in a partially ordered set. Specific formal concepts are selected, similarly to the way in which specific vectors—the eigenvectors of the covariance matrix—are selected when learners are scored on quantitative latent variables. The analogs of scores on a latent variable are the degrees to which learners' performances “display” or “participate in” or “reflect” these specific concepts, which may be thought of as *prototype* or *standards of performance* on the construct. They have the advantage, over hypothesized latent variables whose values are abstracted from observed data, that they are directly expressible in terms of the construct-relevant attributes—that is, in terms of the features of learner's responses to assessment tasks that are taken to be important in a “theory” of “what (good) performance means”, for the educational construct in question. They can be described both by means of their extent (the collection of actual learners' performances exemplifying the concept/standard in question), and by means of their intent [the collection of (fuzzy) attributes that characterizes the standard in question].

### 5.3 Measures and meanings: comparing quantitative and qualitative approaches

Bartl et al. (2018) examined this qualitative factor analytic approach to educational assessment data, with the aims of exploring its applicability in practice, and its application to the study of the construct validity of an examination: the degree to which students' responses, assessed as being at a particular level, matched the intentions of the assessment designers in terms of the qualitative performance standard intended to broadly characterize responses at that level. This is the kind of question that is difficult to study using traditional quantitative methods.

The technical issues involved (for example how to determine the coverage and number of factors that broadly explain the data—analogous to a scree plot in quantitative principal components analysis) will not be rehearsed here. See Bartl et al. (2018) for computational details. For a deeper theoretical treatment of the relationship between eigenvectors (of quantitative covariance matrices) and formal concepts (of qualitative matrices of truth values), see Bradley (2020). The key point is that this approach allows drawing out key features associated with responses assigned to a particular level, by the assessment procedure, and an appraisal of the degree to which each learner's performance on the examination embodies or matches those features. Indeed, it “explained” the data (in terms of proportion of data covered or variance explained) as well as standard principal components analysis, but generated factors exemplifying attributes of performance that seemed to be more easily interpretable.

**Figure 5:**
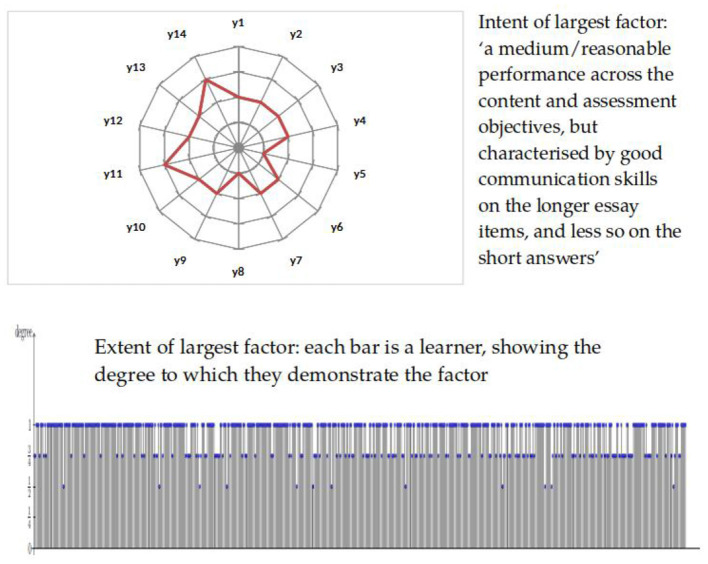
Factor representation for fuzzy data.

Figure 5 shows an example of this, for the educational measurement data studied by Bartl et al. (2018), in which learners were assessed on 14 fuzzy attributes {*y*_1_, …, *y*_14_}, each of which reflected an aspect of the construct, in this case proficiency in the specific subject of “A level Government and Politics”. Each of the attributes corresponds to demonstrating specific types of knowledge and understanding, in accordance with the examiners' agreed understanding of what better/worse proficiency means in this domain. Hence the intent of any given concept can be interpreted by users of the assessment as a description of broadly what that level of proficiency means (and likewise the extent of the concept can be interpreted as an indication of the degree to which each learner has demonstrated that level of proficiency).

The question of the interpretability or explainability of the results of educational measurement procedures—whether those results are numerical scores, or broader grades or levels—is particularly important for high-stakes assessments such as those that underwrite school-leaving qualifications. For learners, clarity about *why* their response to an assessment merited their being characterized as demonstrating a certain level of proficiency is arguably required for reasons of natural justice. For teachers, understanding qualitatively what their students did well, and what they would have to do better to demonstrate more proficiency in a subject domain, is clearly valuable as an input into their future pedagogical practice. Bartl et al. (2018, p. 204) concluded that their approach to qualitative factor analysis yielded “naturally intepretable factors from data which are easy to understand”, but that more research is needed both on technical implementation and on the views of learners and teachers.

### 5.4 Other order-theoretic approaches to educational assessment

In the 1940s Louis Guttman began to develop an approach to psychological measurement (e.g., Guttman, 1944) that led him to think of it as a structural theory (Guttman, 1971), rather than as a process of quantifying amounts of latent traits, and to the development of *facet theory* and *partial order scalogram analysis* (Shye and Elizur, 1994). In the 1980s, Doignon and Falmagne (1999) developed *knowledge space theory*, later evolved into a theory of learning spaces, in which assessment constructs are represented as partially-ordered sets.

Applications of facet theory and knowledge space theory (including related approaches such as Tatsuoka, 2009's *rules space* and Leighton and Gierl, 2007's *cognitive diagnostic models*) normally assume or overlay quantitative latent variable models, to account for “underlying” proficiencies or competencies that determine a learner's progression through such partially-ordered outcome spaces.

However, from the mid 1990s onwards, there has been a strand of research investigating how to extend knowledge space theory to incorporate a focus on skills and competence, leading to the development of *competence-based knowledge space theory* (see e.g., Stefanutti and de Chiusole, 2017). Here, a learner's proficiency or competence is itself conceptualized as a partially-ordered space, rather than a quantity. Ganter and Glodeanu (2014) and Ganter et al. (2017) suggested that formal concept analysis could be applied to study competence-based knowledge space theory, and this is now starting to be done.

For example, Huang et al. (2023) consider how to transform maps from competence-states to “knowledge-states” (types of demonstrated performances) into formal contexts, and hence to represent them as concept lattices. Each node in the lattice then embodies a knowledge-state and a competence-state as its extent and its intent, respectively. This is clearly analogous to the approach set out in Section 4 above.

A very clear application of these methods is to formative, adaptive, assessment and learning systems, where, for instance, they provide an alternative to traditional IRT-based adaptive tests that is more grounded in a theory of learning.

To date there has been less attention to examining summative assessment, and what is often called “educational measurement”, from this perspective. Yet, as argued above, application of non-quantitative approaches needs to be investigated here too, since the pragmatic “as if” approach to routine application of latent variable models is not always justifiable.

## 6 Connections to artificial intelligence

A final reason why it is imperative to pursue research in this area is the rapidly growing application of machine-learning methods, and generative artificial intelligence in particular, in educational contexts. For example, Li et al. (2023) report on using the large language model ChatGPT to score students' responses to (essay style) examinations, and to provide rationales for the scores awarded.

Because the outputs of generative AI applications using large language models are no more than statistically plausible sequences of words, albeit expressed in well-formed natural language, their validity, fairness and reliability is hard to establish theoretically. That is because they are produced using so-called *subsymbolic* approaches to AI (see e.g., Sudmann et al., 2023), such as deep neural nets, rather than *symbolic* methods that aim to use forms of explicit logical inference to arrive at results: analogously to reasoning about a learner's response to a task with reference to criteria for betterness that define the kind of proficiency one intends to measure by administering the task.

An interesting angle opened up by the qualitative measurement approach described above is the possibility of combining formal concept analysis with neural networks to enhance the explainability of, for example, scores derived from applying a classifier based on a large language model to learners' performances on an examination.

Some initial work in this area has been done by Hirth and Hanika (2022) and Marquer (2020), among others. This kind of analysis could complement quantitative approaches to explaining marks or scores awarded to learners' responses, such as dimension-reduction of the high-dimensional vector space that the language model uses to represent linguistic artifacts—such as learners' responses to assessment tasks—as numerical vectors. In fact, Bradley et al. (2024) have recently shown that there is a relationship between quantitative techniques based on linear algebra, such as latent semantic analysis, and formal concept analysis, such that the latter can be seen as a more general form of the former. They have applied formal concept analysis to elucidating how semantics appears to arise from syntax, and to study the structure of semantics, when large language models are used to produce outputs from qualitative data.

Clearly, the practice of educational (and psychological) measurement is changing as technology changes. Tasks can be administered digitally; the widespread availability of devices with reasonable processing power means the possibilities for task design are much more open than they were a decade ago, and they will continue to evolve. The data that is gathered about learners, given their responses to these tasks, can be more unstructured than category-labels or scores: it may be text, audio, or video, and/or representations of such data for example in a vector-space language model. To the extent that human assessors form part of measurement procedures, for example to apply scoring rubrics, they may be partially or wholly replaced by AI.

What remains fundamental, however, is the need to base these measurement procedures in a theory of what defines or constitutes better or worse proficiency, in the domain of interest, and hence what substantive and semantic content is entailed in statements such as “this learner got a score of 137”, or “this learner has 1.07 logits of proficiency”; or “this learner has demonstrated three of the four prototypical aspects of proficiency that define a “grade B standard”, or whatever — what it means to locate them, via a measurement, at a certain position in a (quantitative or other) space.

## 7 Discussion

### 7.1 Qualitative educational assessment is possible in principle, and includes quantitative measurement as a special case

This paper has argued that it is not warranted to assume the phenomena studied in psychometrics, and in educational measurement in particular, are necessarily appropriately conceptualized as quantities. In cases where an assumption of quantitative structure *is* appropriate, then measuring an instance of such a phenomenon means locating it at a point on the real continuum. In cases where the assumption is not appropriate, the idea of measurement becomes, more generally, locating the measurand in a suitable logical space, that is defined in a way that is relevant for the phenomenon.

When the measurand is quantitative and the logical space is the real numbers, the usual methods of psychometric analysis for estimating latent parameters can be deployed. But, *contra* Thurstone (1928), the paper has argued that it is not necessary to “force” theoretically well-supported constructs into a more reductive quantitative form if that is not appropriate. Hence the argument of this paper is not that psychometrics should be replaced, but that its repertoire of measurement approaches should be widened to cope with measurands that are intrinsically non-quantitative in nature.

The paper suggests that the outcomes of educational measurement procedures can be thought of, in general, as fuzzy relational systems; and that fuzzy formal concept analysis is an appropriate tool to describe data models for the measurands they aim to locate. These models instantiate the “betterness” relation for the measurand: they model the notion of “what good performance looks like”. Such an account or understanding is prior to, and necessary for, an understanding or agreement as to “what being (more or less) proficient” means, in an educational domain. It forms the theory of the construct (one might say, the theory of *value* for the construct, and hence a foundation for evaluation of construct *validity*).

### 7.2 Educational constructs are contestable, intersubjective, temporally-located phenomena

These theories of constructs such as proficiency or competence in a domain are necessarily contestable, intersubjectively constructed, and liable to change over time. Intersubjectivity (Chandler and Munday, 2011) refers to the mutual construction of relationships through shared subjectivity. Things and their meanings are intersubjective, within a given community, to the extent that the members of the community share common understandings of them. Thus, the community that constitutes the competent authority for defining an educational construct decides what particular knowledge, skills, and understanding it will encompass, and what will count as better or worse configurations of these aspects as possible ways of being proficient in the domain in question. Thus, for instance, the job of someone marking responses to an examination that is designed to measure that construct is to apply the mutually constructed and agreed standard consistently to each response she marks (irrespective of whether she personally agrees that it is the “right” standard).

We do not have to think of data models that encode these intersubjective constructions as (more or less accurate) representations of some objective or underlying “true” account of the measurand in question. As van Fraassen (2008, p. 260) notes, “in a context in which a given model is *someone's* representation of a phenomenon, there is **for that person** no difference between the question *whether a theory fits that representation* and the question *whether that theory fits the phenomenon*.”

### 7.3 More research is needed on using partial orders in practice, on linking different assessments of the same construct, and on fuzzy valuations

Section 4 argued that in general the data models for measurands such as proficiency in an educational domain are partial orders. This perhaps goes against a relatively strongly ingrained concept of educational assessment as synonymous with *ranking* (e.g., Holmes et al., 2017). Yet in many cases, once a theory of (betterness for) a construct has been settled, rankings are neither necessary nor needed. Two learners' proficiency values may simply be qualitatively different (non-comparable). For instance in Figure 2, this is the case for learners 3 and 6. But both learners 3 and 6 have performed better than learner 1. So if learner 1's performance was sufficient to merit a “pass” grade, let us say (or was picked out as a “pass” grade prototype), then we know that learners 3 and 6 are also sufficiently proficient to be awarded a pass, even though it is not meaningful to say that their actual demonstrated proficiencies were the same, or that either one is more or less proficient than the other. More work is needed on the scope for using visualizations such as concept lattices to help educational assessment designers and teachers engage with and interrogate the outcomes of educational measurement procedures (see, for a start, Bedek and Albert, 2015).

A common application of quantitative latent variable models is to *equating* or *linking* different forms of tests of learners' proficiency in a certain domain. Typically, equating studies are designed to answer questions like “what score on form *X* of a test is equivalent to (represents the same level of proficiency as) a given score on form *Y* of the test?”. In practical applications in many educational contexts however, such as grading students' responses to school-leaving examinations (Newton et al., 2007), one is not so much interested in constructing a monotone map from scores on *X* to scores on *Y*, as in ensuring that the levels or kinds of proficiency demonstrated by students graded, say, A, on this year's examination, are “equivalent”, or “of a comparable standard” to the type of proficiency demonstrated by students graded A on last year's examination.

An area for further research is how to implement such comparability studies in the fuzzy-relational approach to educational assessment proposed in this paper. For example one could take the students graded A on each of the two forms of an assessment, and examine the intents of the formal concepts that form their largest factors (cover an appreciable proportion of the data, in the terms of Bartl et al., 2018). Are these sufficiently similar to count as equivalent demonstrations of proficiency, and what criteria should be applied to appraise similarity?

A deeper question is how the truth degrees that summarize each learner's demonstration of each construct-relevant attribute are determined. In some cases this is straightforward in practice (e.g., for dichotomously-classified test items such as multiple-choice questions); but when judges are needed as part of the measurement procedure, different judges may give different truth values, so what counts as a reasonable or acceptable value? A full account of this aspect of qualitative valuation may need to draw on *rough fuzzy logic* (Dubois and Prade, 1990; Bazan et al., 2006), itself an active area of research in machine learning. Certainly more research is needed here.

Having said that, there is strong support for connecting fuzzy relational structures to cognitive theories of concept formation, when exploring the question of how experts—and these days, AIs—learn to categorize (value) responses to tasks, given some prototypical exemplars: see for example Bělohlávek and Klir (2011).

The outcomes of educational measurement procedures are ultimately underpinned by value judgements about exactly what to assess and how to assess it. As Wiliam (2017, p. 312) puts it: “whereas those focusing on psychological assessment tend to ask, ‘Is this correct?', those designing educational assessment have to ask, ‘Is this good?”'. So questions about how to use mathematical methods in these contexts, in a way that leverages their power, but is not unduly reductive, will no doubt always be debated. It is hoped this paper makes a helpful contribution to that debate.

## Data Availability

The raw data supporting the conclusions of this article will be made available by the authors, without undue reservation.

## Footnotes

^1^See the Appendix for definitions of *total order* and *quantity*. Informally, a totally ordered set *X* is one in which all the members can be ranked—there is an ordering ≥ such that either *x*≥*y* or *y*≥*x*, for all *x* and *y* in *X*. A property is a quantity if its values are totally ordered and also additive—that is, they can be combined in a way that mirrors the properties of the addition of numbers. Additivity is required for a property's values to form an *interval scale* or a *ratio scale*, in the terminology of Stevens (1946). A quantitative property is *continuous* if its possible values form a continuum with no “gaps”.

^2^See the Appendix for a formal definition of *partial order*. In essence, when entities are partially ordered, there may exist pairs of entities that are not directly comparable, and the entities cannot necessarily be placed in a single linear sequence (a ranking) with respect to the feature of interest. In educational tests, each individual item (question or task) typically totally orders the respondents with respect to that item (for example “those who got the question right” ≥ “those who got the question wrong”; or “those who scored 3 marks” ≥ “those who scored 2 marks” ≥ “those who scored 1 mark' ≥ “those who scored 0 marks”) In general, however, the joint result (the product) of all of these total orders is an overall partial ordering of respondents, with some patterns of item responses not being directly comparable with others.

^3^A further extension of Heilmann's position would be to consider mappings from a QRS to another QRS: in other words, to relax the restriction that the “representing” structure should be numerical. Such a generalization might permit both RTM and van Fraassen's approach to be located, from a formal mathematical perspective, within the general theory of structure known as category theory, but will not be pursued here.

^4^The Rasch model, also known as the 1-parameter item response model, postulates that the log-odds of a test-taker of ability θ correctly answering an item of difficulty δ is simply θ−δ (in the case of a test consisting of a sequence of dichotomously-scored items). There are of course other item response models that postulate additional item parameters, but Rasch theorists hold that the 1-parameter model is theoretically more appropriate as a basis for enabling measurement because it enables, within a given collection of persons and items, so-called invariant comparisons of persons (as to their ability) and items (as to their difficulty): see Andrich and Marais (2019, p. 80).

^5^The development of quantum theory in the twentieth century problematized the classical epistemological viewpoint on measurement as “discovery”. As Peres (1995, p. 14) observes, “classical physics assumes that the property which is measured objectively *exists* prior to the interaction of the measuring apparatus with the observed system. Quantum physics, on the other hand, is incompatible with the proposition that measurements discover some unknown but pre-existing reality.”

^6^Algebras, lattices, and posets (short for partially-ordered sets) are types of mathematical structures. In particular, a lattice is a partially-ordered set (see the Appendix for a definition) in which each pair of elements has a least upper bound and a greatest lower bound.

^7^Normally concept lattices are drawn as so-called Hasse diagrams with the least general concept at the bottom, and the most general concept at the top. An arrow is drawn upwards from concept *A* to concept *B* if *B* is more general than *A*. In the educational assessment context, we naturally regard the best performance as the *top* concept, which means we need to reverse the usual ordering (in mathematical terms, we use the *dual* lattice). This is done throughout this paper, for example in Figure 2, where the worst level of proficiency (exhibited, to degree 0.5, by learner *l*_5_) is at the bottom of the diagram, and the best level (exhibited by learners *l*_2_, *l*_3_, *l*_4_, and *l*_6_, also to degree 0.5) is at the top.

^8^As Weyl (1952, p. 8) noted, “For measurement the distinction is essential between the ‘giving' of an object through individual exhibition on the one side, in conceptual ways on the other”.

^9^As Michell (2009) observes, “Tabulated numbers are shorthand for a set of propositions that tell where the numbers came from. Furthermore, deductions from a data set are inferences from these propositions.”

^10^Part of paper 1 of the AQA A level physics examination taken in 2018. Unusually for an A level assessment, the items here are all dichotomous (multiple-choice questions). The lattices would be even larger if the items admitted fuzzy valuations.

^11^The product of two real-valued matrices *A* and *B* is defined by setting its (*i, j*) entry (*AB*)_*ij*_ to the inner product of row *i* of *A* with column *j* of *B*: i.e., ${\left(AB\right)}_{ij}:=\overset{k}{\underset{p=1}{\sum }}A_{ip}B_{pk}$. When the matrix entries are truth values, they are elements of a type of lattice that is equipped with an operation ⊗ to combine values. In this case the matrix product *A* ◦ *B* is defined as ${\left(A◦B\right)}_{ij}:=\vee_{p=1}^{k}A_{ip}⊗B_{pk}$, where ∨ is the supremum over the indicated set (see Appendix).
