# Supplementary material for: Educational assessment without numbers
Source: Front Psychol. 2024 Oct 2;15:1399317. doi: 10.3389/fpsyg.2024.1399317 (PMC11480064; doi:10.3389/fpsyg.2024.1399317)
Supplement: Supplementary file 1 [file Table_1.pdf]

APPENDIX - DEFINITIONS

Order relations

Let X be a set. A *binary relation* R on X is a collection of ordered pairs of elements of X. We usually write xRy, rather than $(x, y) \in R$, if x is related to y by R. The relation R is said to be:

- *reflexive* if xRx for all $x \in X$;
- *transitive* if for all $x, y, z \in X$, whenever xRy and yRz, then xRz;
- *anti-symmetric* if for all $x, y \in X$, if xRy and yRx, then $x = y$; and
- *strongly connected* if for all $x, y \in X$, either xRy or yRx.

A binary relation that is reflexive, transitive, and anti-symmetric is called a *partial order*. A *partially-ordered set*, or *poset*, is a set with a partial order on it. A partial order that is, in addition, strongly connected is called a *total order*.

An alternative way of defining a partial order is as a directed graph (collection of objects and arrows), in which there is at most one arrow between any two objects. So when a set of objects X is partially ordered by a relation $\succeq$, we can use '$a \leftarrow b$' as an alternative to '$a \succeq b$', for $a, b \in X$. A total order is the special case in which there is exactly one arrow between any two objects. Hasse diagrams, such as Figures 2 and 3, depict posets in this way. The Hasse diagram of a total order is a line (a ranking).

Lattices

Let $(L, \succeq)$ be a poset, and let S be a subset of L. An *upper bound* for S is an element $u \in L$ such that $u \succeq s$ for all $s \in S$. u is the *least upper bound* or *supremum* of S, denoted if $y \succeq u$ for all upper bounds y of S. Dually, a *lower bound* for S is a $l \in L$ such that $s \succeq l$ for all $s \in S$, and l is the *greatest lower bound* or *infimum* of S if l for all lower bounds x of S.

A *lattice* is a poset for which every two-element subset x, y has a supremum $x \vee y$ and an infimum $x \wedge y$. A *complete lattice* has a *bottom* (least) element, usually denoted 0 or $\perp$, and a *top* (greatest) element, denoted 1 or $\top$.

Quantitative structure

Let P be a property of interest. For example, P could be the mark-level (e.g. a mark out of 25) given to an English essay by an examiner. P could be the age of a student, the length of a rod, or the colour of an object. In each case, P can assume, for any particular object in its domain, one of a finite or infinite collection of labels, descriptions, or values drawn from a set V.

Suppose there is a total-order relation $\geq$ on the possible values V of P. Suppose, also, that there is a binary operation + on V (i.e. a mapping + that assigns to each pair of values (u, v), with $u, v \in V$, another value $u + v \in V$) that satisfies the following six conditions. For any $x, y, z \in V$:

- (associativity) $x + (y + z) = (x + y) + z$;
- (commutativity) $x + y = y + x$;
- (monotonicity) $x \geq y$ if and only if $x + z \geq y + z$;
- (solvability) if $x \geq y$ but $x \neq y$, then there exists a $z \in V$ such that $x = y + z$;
- (positivity) $x + y \geq x$;
- (Archimedean condition) there exists a positive integer n such that $nx \geq y$, where the notation 'nx' means $x + x + ... + x$ (n times).

Then P is said to be a *quantity*. A *continuous quantity* Q is a quantity whose possible values form a continuum with no 'gaps'. Formally, this requires two further conditions. Firstly, Q must be *dense*, which means that between any two values or levels of Q, there is another value:

- (denseness) if x and y are values of Q, with $x \geq y$, then there exists a value z of Q such that $x \geq z \geq y$.

Secondly, Q must be *complete*, which means that all sets of values of Q that are bounded above have a least upper bound:

- (completeness) Let X be a set of values of Q. Then there is a value y of Q such that (i) $y \geq x$ for all $x \in X$; and (ii) if z is any other value such that $z \geq x$ for all $x \in X$, then $y \geq z$.
